# An optical biosensor assay for rapid dual detection of Botulinum neurotoxins A and E

**DOI:** 10.1038/srep17953

**Published:** 2015-12-09

**Authors:** Christian Lévêque, Géraldine Ferracci, Yves Maulet, Christelle Mazuet, Michel R. Popoff, Marie-Pierre Blanchard, Michael Seagar, Oussama El Far

**Affiliations:** 1 INSERM, UMR_S 1072, 13015 Marseille, France; 2 CNRS, UMR 7286, Plate-Forme de Recherche en Neurosciences PFRN, 13015 Marseille, France; 3 Aix-Marseille Université, 13015 Marseille, France; 4 CNR Bactéries Anaérobies et botulisme, Unité des Bactéries anaérobies et toxines. Institut Pasteur, 28 rue du Dr Roux, 75724 Paris Cedex 15, France

## Abstract

The enzymatic activity of the pathogenic botulinum neurotoxins type A and E (BoNT/A and E) leads to potentially lethal paralytic symptoms in humans and their prompt detection is of crucial importance. A chip assay based on Surface Plasmon Resonance monitoring of the cleavage products is a simple method that we have previously established to detect BoNT/A activity. We have now developed a similar format assay to measure BoNT/E activity. A monoclonal antibody specifically recognizing SNAP25 cleaved by BoNT/E was generated and used to measure the appearance of the neo-epitope following injection of BoNT/E over SNAP-25 immobilized on a chip. This assay detects BoNT/E activity at 1 LD_50_/ml within minutes and linear dose-responses curves were obtained using a multiplexed biosensor. A threshold of 0.01 LD_50_/ml was achieved after 5 h of cleavage. This assay is 10-fold more sensitive than the *in vivo* assay for direct detection of BoNT/E in serum samples. The SNAP25 chip assay is able to discriminate in an automated manner the presence of BoNT/E, BoNT/A or a combination of both toxins.

Botulinum neurotoxins (BoNTs) constitute a family of 7 serologically distinct neurotoxins (from A to G) produced by *Clostridium botulinum* and a few other species of *Clostridium*. BoNT/ A, B and E are the principal causes of human botulism. Toxicity occurs mainly after oral ingestion of contaminated food. BoNT’s escape the gastro-intestinal tract and a small percentage enters the bloodstream. Although BoNT’s found in human sera are in the low fmolar concentration range, specific receptors localized in the peripheral nervous system concentrate the toxin, mediate its neuronal internalization and the consequent disabling of synaptic transmission^1^. These potent neurotoxins are also considered as one of the most toxic bioweapons constituting a threat for the public^2^.

Botulism can be effectively treated immunologically using an equine trivalent antitoxin^3^. However, rapidity is a crucial issue in BoNT diagnosis since anti-toxin antibodies become ineffective when the toxin penetrates into neurons and no antidote is available. The decision to use serotherapy is often taken on the basis of clinical symptoms and the most direct way to subsequently confirm diagnosis is to identify the BoNT type in the patient’s serum and/or stool using the standard mouse median lethal dose LD_50_ method^4^. The specificity of this *in vivo* assay lies in the absence of symptoms when specific anti-BoNT antibodies are co-injected with the experimental sample. However, the *in vivo* method is not really adapted for rapid molecular diagnosis since several hours or days (range 6–96 h) are necessary for toxin type identification, especially for BoNT concentrations that cause symptoms but not death^5^. Moreover this procedure is cumbersome and ethically questionable. Hence *in vitro* methods allowing rapid and sensitive detection of BoNTs have been developed.

Type E botulism affects humans and animals^6^. Intoxication is particularly associated with ingestion of marine products as strains producing type E toxin are frequently found in fish and aquatic environments mainly from the Northern countries of North hemisphere^7^,^8^. BoNT/E intoxication is more rarely associated with other foods such as ham^9^.

BoNT/E is synthetized as a single chain molecule of 150 kDa associated with non-toxic proteins. It acquires higher toxicity upon nicking with an exogenous protease, such as trypsin, at about one third of the length from the N-terminus^10^,^11^. The resulting activated protein consists of a light (50 kDa) and heavy chain (100 kDa) linked by a disulfide bond and non-covalent interactions. The BoNT/E heavy chain controls cellular internalization via binding to the synaptic vesicle protein SV2 through receptor-mediated endocytosis and translocation^12^,^13^. After translocation into the cytoplasm, the light chain of BoNT/E targets Synaptosomal-associated protein of 25 kDa (SNAP-25), a protein associated with the inner leaflet of the plasma membrane which is involved in the formation of a multi-protein complex that generates a driving force for exocytosis. BoNT/E cleaves off a C-terminal 26 amino acid fragment of the SNAP-25 inducing a persistent but reversible inhibition of neurotransmission. BoNT/A and BoNT/C also target SNAP-25, but cut the protein at distinct cleavage sites^1^.

Measurement of BoNT/E enzymatic activity constitutes the most sensitive approach to specifically detect this toxin. A variety of *in vitro* activity assays have already been published including methods using capillary gel electrophoresis^14^, ELISA^15^, mass spectrometry^16^,^17^ or fluorescent and electrochemical readouts^8^,^18^,^19^.

Since the 1990s, Surface Plasmon Resonance (SPR) has proven to be one of the most versatile biophysical methods for biosensor applications in the field of biology, biomedicine and biochemistry as well as food safety. Optical SPR biosensors operate in a label-free manner by measuring, as molecules bind in real time, the change in the refractive index within the evanescent field on the apparatus surface. We have recently developed a new SPR-based method to rapidly assay BoNT/A enzymatic activity with very high sensitivity^20^,^21^. In this paper, we describe, using this integrated optical “on-chip” method, the development of an assay able to detect simultaneously the presence of BoNT/A and BoNT/E.

## Results

### Production of a monoclonal antibody recognizing the neo-epitope generated by BoNT/E

**Figure 1:**
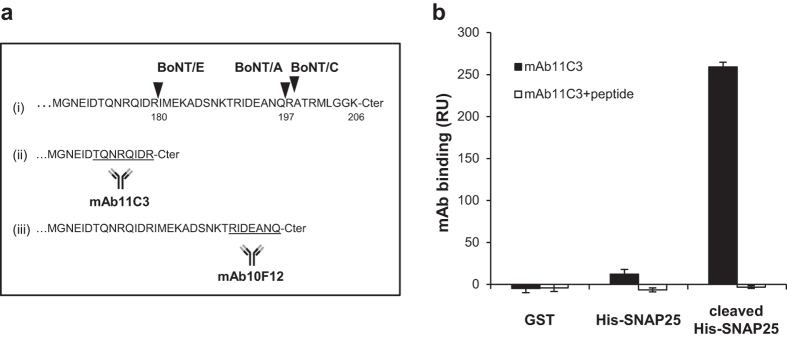
Specificity of mAb11C3 antibody. (**a**) Sequence of the C-terminal domain of SNAP-25 with cleavage sites for BoNT/A, BoNT/E and BoNT/C (arrowheads in “i”). The new C-terminal domains after BoNT/E (“ii”) and BoNT/A (“iii”) cleavage are illustrated. The peptide sequences used to generate mAbs 11C3 and 10F12 are underlined. (**b**) His-SNAP25 was treated or not with BoNT/E and immobilized by amine coupling on a sensor chip. mAb11C3 (10 μg/ml) was then injected for 2 min and the amount of cleaved-SNAP25 measured (±SD, n = 3 mAb injections).

Mice were immunized using an eight residue peptide (amino acid 173–180) corresponding to the new C-terminal domain of SNAP-25 produced by the proteolytic activity of BoNT/E (Fig. 1a). Hybridomas were generated and several clones were selected by ELISA for recognition of the immunization peptide. One clone (mAb 11C3) was selected and characterized as follows: upon injection in the mobile phase, strong binding to immobilized His-SNAP25 previously cleaved by BoNT/E was measured, whereas no binding was detected either to an irrelevant control protein (GST) or to uncleaved His-SNAP25 (Fig. 1b). The signal was abolished if mAb11C3 was injected with an excess of the cognate peptide (Fig. 1b). In order to verify the specificity of mAb11C3 for recognition of a native substrate cleaved in a cellular context, we intoxicated neuronal cultures with BoNT/E. Immunofluorescence staining using BoNT/E-treated versus untreated neuronal cultures showed that mAb11C3 recognized only BoNT/E treated neurons (Fig. S1).

### On-chip substrate cleavage by BoNT/E: assay principle

**Figure 2:**
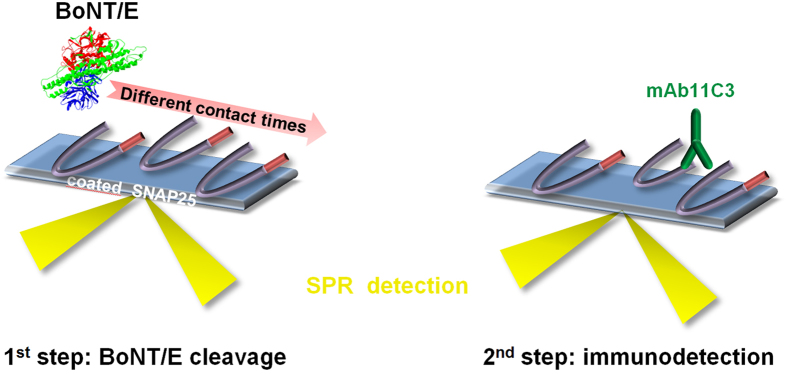
Scheme illustrating the general principle of the assay. In the first step, a BoNT/E sample is injected over the SNAP-25 sensor chip for a pre-determined contact time. In the second step, the neo-epitope produced by BoNT/E activity is detected using mAb11C3. Regeneration with a short pulse of acidic buffer (not illustrated), triggers dissociation of bound antibodies and allows a new cycle of cleavage/detection.

This assay is based on the measurement of the catalytic activity of BoNT/E using an SPR biosensor, integrating all steps on a chip. Figure 2 shows the basic two-step principle of our approach. The first step consists in injecting BoNT/E at a given concentration for a controlled period of time over a sensor surface coated with His-SNAP25. BoNT/E cleaves the peptide bond between residues 180–181, thus generating a new SNAP-25 C-terminus. In the second step, mAb11C3 antibody is injected and the extent of cleavage is quantified by monitoring the increase in Resonance Units (RU), reflecting antibody binding. We found that mAb11C3 binding was reproducible when the antibody injection was repeated after regeneration by acidic pH (CV = 3–4%, n = 14).

### Assay specificity

**Figure 3:**
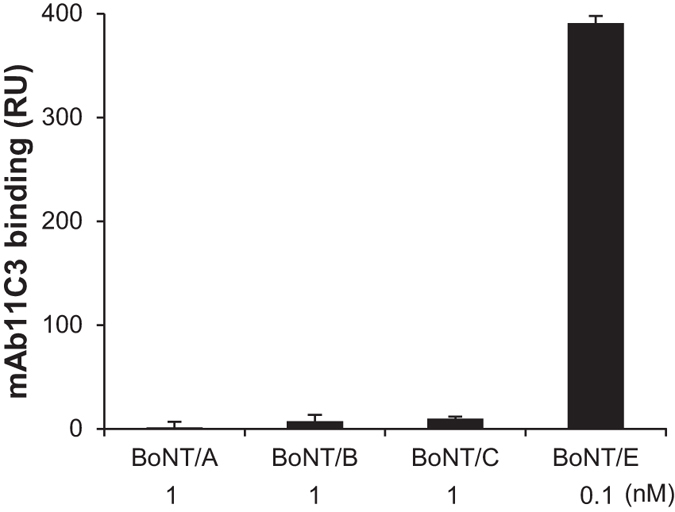
Assay specificity. Samples containing BoNT/A, B, C or E were injected for 10 min at the indicated concentrations over a SNAP-25 sensor chip. The binding signal of mAb11C3 to all flowcells was then compared. (±SD, n = 3 mAb injections).

In order to address the BoNT serotype specificity of the assay, BoNT/A, B, C or E were injected over independent sensor chip flow cells coated with His-SNAP25. BoNT/A and C cleave SNAP-25 at different sites than BoNT/E (Fig. 1a) and therefore generate different C-termini. Consequently their proteolytic products should not be recognized by mAb11C3. BoNT/B cleaves the protein VAMP2 and has no proteolytic activity on SNAP-25^1^. As shown in Fig. 3, mAb11C3 bound neither to SNAP-25 cleaved by high concentrations of BoNT/A and C (1 nM) nor to BoNT/B (1 nM) -treated flow cells. In contrast mAb11C3 strongly bound to BoNT/E-treated SNAP-25 (0.1 nM). This result established that mAb11C3 can specifically detect BoNT/E catalytic activity using an “on-chip” SPR-based assay.

### Dose and time dependence of SNAP-25 BoNT/E cleavage

**Figure 4:**
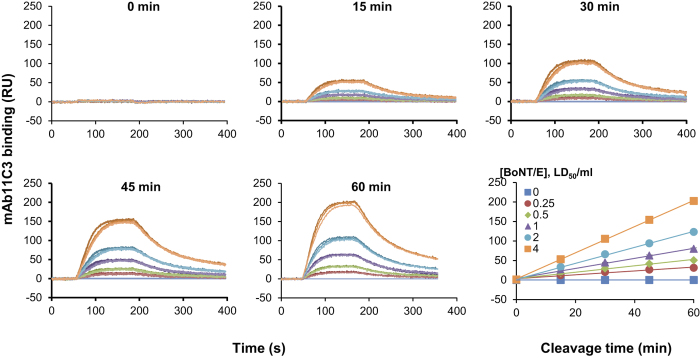
Dose-dependence and kinetics of the BoNT/E assay. After His-SNAP25 immobilization on 6 independent spots, six samples with the indicated BoNT/E concentrations were injected for 4 sequential 15 min periods (ie. to yield 15, 30, 45 and 60 min cumulated cleavage times). At the end of each 15 min injection, detection of BoNT/E enzymatic activity was achieved by injecting mAb11C3 (n = 3). Each panel represents superimposed sensorgrams obtained for mAb11C3 binding at the indicated times. The last panel illustrates cleavage kinetics at each concentration. The figure is representative of three independent experiments. Note that error bars (SD) are included but too small to be distinguished.

We then explored the time-and dose-dependence of the SPR-based assay. We used a multiplexed biosensor (ProteOnXPR36) which incorporates six parallel flow channels^22^. In order to evaluate how these *in vitro* results compare to the mouse bioassay, BoNT/E concentrations were expressed in mouse lethal dose units (LD_50_/ml). Experiments were carried out to measure mAb11C3 binding after repeated injections, for a limited contact time (15 min) of a BoNT/E concentration range (0.25–4 LD_50_/ml). Although no signal was measured before BoNT/E injection (Fig. 4, 0 min), after 15 min of cleavage, specific mAb11C3 binding was detected only at the highest BoNT/E concentrations used (4, 2 and 1 LD_50_/ml) (Fig. 4, 15 min). Additional rounds of BoNT/E injections were further performed (15 min each) separated by an acidic regeneration of the chip. As early as the second BoNT/E injection round (cumulated contact time 30 min), a specific mAb11C3 binding signal was observed even with the lowest (0.25 LD_50_/ml) toxin concentration (Fig. 4, 30, 45 and 60 min). Binding responses at plateau values were plotted and found to be dependent on cleavage-time (Fig. 4 last panel). For all BoNT/E concentrations in this time scale, curves were linear, indicating that the reaction is not substrate-limited. In addition, the regeneration step did not affect subsequent cleavage steps on the same functionalized surface (Fig. 4). Thus, a specific feature of this SPR procedure is that the His-SNAP25 chip can be reused for several assays provided that low BoNT/E concentrations are used, in order to avoid substrate depletion. However, we noticed that prolonged cleavage times and/or higher BoNT/E concentrations can induce a loss in linearity due to substrate depletion (Fig. S2).

### Rapid detection of BoNT/E activity at 1 LD_50_/ml

Using a ProteOn biosensor, BoNT/E proteolytic activity was easily detectable by mAb 11C3 at 1 LD_50_/ml (22 pM) after only 15 min of cleavage (Fig. 4 and Table 1). The rapidity of detection at 1 LD_50_/ml was confirmed using a Biacore T200 biosensor, on which experiments can be implemented more quickly, and which has a slightly higher sensitivity to ProteOn biosensor due to a lower background. In this case, BoNT/E activity at 1 LD_50_/ml was detected after 5 minutes of contact time (Table 1).

**Figure 5:**
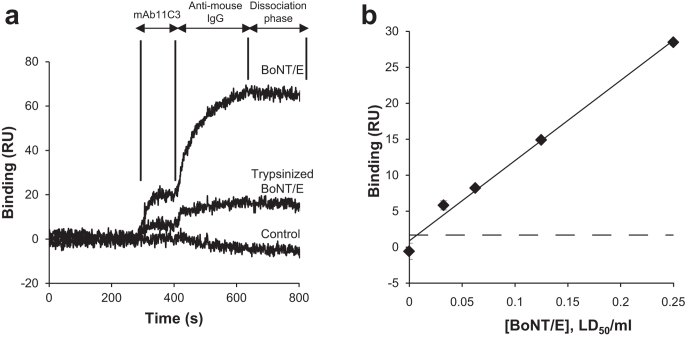
Assay sensitivity for short and long cleavage times. (**a**) Cleavage buffer (Control), 1 LD_50_/ml BoNT/E (22 pM) and 1 LD_50_/ml trypsinized BoNT/E (360 fM) samples were injected for 15 min over different SNAP25 spots. Tandem injection of mAb11C3 and anti-mouse Fc antibody was used to amplify the detection of cleavage products. Traces represent a typical experiment with superimposed signals generated by antibody injections. (**b**) Trypsinized BoNT/E samples (0.03, 0.06, 0.125 and 0.25 LD_50_/ml) were injected over different SNAP25 spots for 5 hours. Co-injection of mAb11C3 and anti-mouse Fc antibody was used to detect cleavage products. Data are means of mAb binding signal (n = 3 ± SD). Dashed line denotes the mean background plus three SD (LOD) obtained in the absence of BoNT/E. Representative of three independent experiments.

It has been reported that trypsinization of BoNT/E induces an increase in its toxicity in the mouse *in vivo* bioassay^10^. In fact, we found that trypsinization of BoNT/E led to a 60 fold increase in potency in the mouse bioassay (data not shown). However, based on their respective LD_50_ determinations, the enzymatic activity of trypsinized BoNT/E at 1 LD_50_/ml (5.8 ± 1 RU, n = 5) was found to be a third of that of the non trypsinized form (16.3 ± 2.7 RU, n = 5, Table 1), as illustrated in Fig. 5 (mAb11C3).

In order to increase SPR responses, a secondary anti-mouse IgG antibody was used to amplify the mAb11C3 signal via the co-inject mode of the SPR instrument. This automated procedure allows consecutive loading of both mAb11C3 and the secondary antibody in the injection loops. As shown in Fig. 5a, the secondary antibody binding response took place immediately after the end of mAb11C3 injection. This amplification mode provided a 3–4 fold increase in RU responses (Table 1 and Fig. 5). Moreover the secondary antibody stabilized the binding of the primary one, as the dissociation step was significantly slowed (compare Fig. 4 and Fig. 5a). This amplification step provided thus an efficient means to amplify binding signals and to detect trypsinized or non trypsinized BoNT/E activity at 1 LD_50_/ml in 15 minutes.

### Limit of sensitivity in absence and presence of serum

As the sensitivity is a function of cleavage time, we explored the lowest BoNT/E concentration that allowed detectable signals after 5 h of contact with the substrate. Five BoNT/E concentrations (0.03 to 0.25 LD_50_/ml) were injected and the cleavage products detected using mAb11C3 in tandem with secondary antibody to maximize sensitivity. The dose-response relationship at these very low BoNT/E concentrations was linear (Fig. 5b). The SPR-based assay exhibited a limit of detection of 0.01 LD_50_/ml for BoNT/E and 0.03 LD_50_/ml for trypsinized BoNT/E (Fig. 5b and Table 1).

We then evaluated the performance of the SPR assay with trypsinized BoNT/E spiked in serum. In preliminary experiments with a 15 min cleavage time, we had found that diluting BoNT/E in cleavage buffer containing 10% serum did not affect assay sensitivity (11.4 +/− 2 RU (n = 3) without serum versus 9.8 +/− 3 RU (n = 4) at 2 LD_50_/ml with serum). This result indicated that BoNT/E could be directly detected in serum, provided that the specimen is diluted 10-fold before injection. To investigate the *in vitro* assay sensitivity in presence of serum, and to ensure the robustness of the SPR method using another source of toxin, crude BoNT/E complex was produced and healthy serum samples spiked with different amounts of toxin. We then compared in parallel the sensitivity of the mouse bioassay and the SPR assay. Aliquots were either administrated in mice without dilution (1 ml/mouse) or injected for 5 hours on the biosensor after 10 fold dilution in cleavage buffer. As indicated in Table 2, no mouse lethality was observed when using toxin concentrations below 1 LD_50_/ml. The activity of BoNT/E concentrations as low as 0.125 LD_50_/ml was still robustly detected using SPR. Thus, using BoNT/E samples in undiluted serum, the *in vitro* test was nearly 10 times more sensitive than the *in vivo* assay. Altogether these results indicate that the SPR assay is about 100 fold more sensitive than the *in vivo* assay in the absence or in the presence of 10% serum detecting as low as 0.01 LD_50_/ml.

### Co-detection of BoNT/E and BoNT/A activity

**Figure 6:**
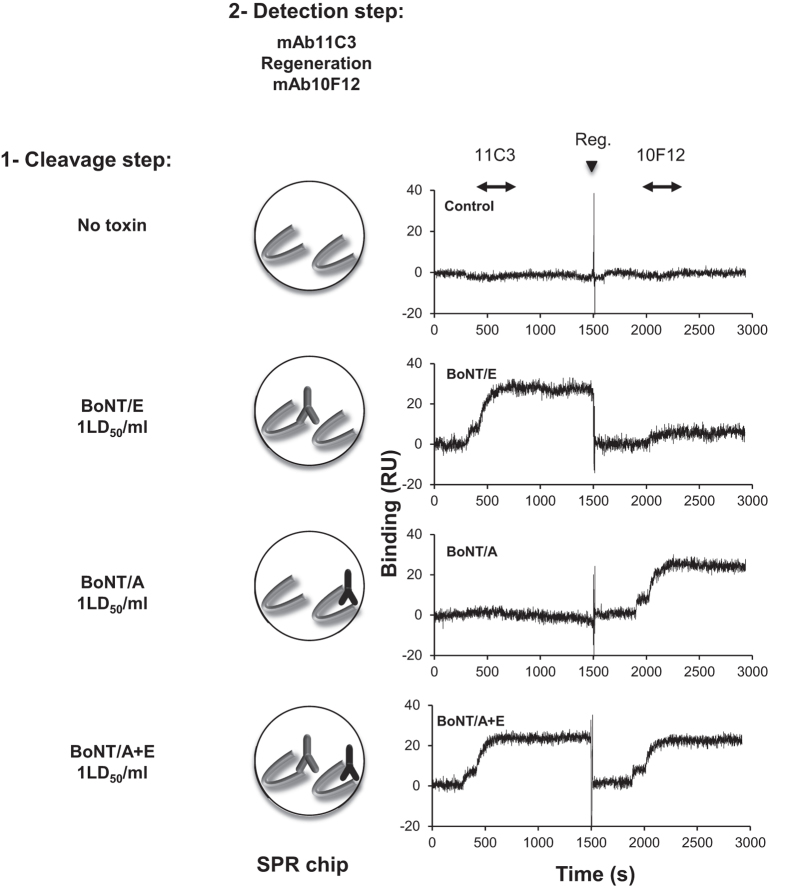
Co-detection of BoNT/A and BoNT/E activities. BoNT/A (1 LD_50_/ml), trypsinized BoNT/E (1 LD_50_/ml), a mixture of both toxins (1 LD_50_/ml each) or BoNT-free buffer were injected in parallel for 15 minutes on 4 SNAP-25 spots. BoNT/E activity was revealed using mAb11C3 and anti-mouse Fc antibodies. BoNT/A activity was revealed using mAb10F12 and anti-mouse Fc antibodies. Rapid treatment with pH 2.0 buffer (reg) was used between mAb11C3 and mAb10F12 detection to regenerate the surface. Representative of 3 independent experiments.

We have previously reported the use of a SNAP25 chip to assay BoNT/A activity^20^ using a monoclonal antibody (mAb10F12), that specifically recognizes the epitope generated on SNAP25 by BoNT/A activity (Fig. 1a). In an attempt to establish a dual SPR assay, we used both mab10F12 and mAb11C3 to verify whether our SPR method could discriminate between the activities of the two toxins and if it can detect the presence of both toxins in the same sample. BoNT/E and BoNT/A cleave SNAP-25 at different sites, separated by 17 residues, in the C-terminal domain of the protein (Fig. 1a). BoNT/E activity removes the BoNT/A cleavage site (Fig. 1a) and therefore could in principle hinder the development of a multiplexed assay. Consequently, BoNT/E activity should be measured on a substrate chip after treatment with BoNT/A, but the opposite should theoretically not be possible. In order to overcome this problem, we used low concentrations of toxins (1 LD_50_/ml), at which a single substrate molecule has little probability of being cleaved by both toxins. We set up a protocol where BoNT/E, BoNT/A or a mixture of both toxins were injected for 15 minutes on different flow cells of a ProteOn apparatus. The cleavage activity of each toxin was then followed by consecutive injection of mAb11C3 and mAb10F12 for detection of BoNT/E and BoNT/A activity respectively. As shown in Fig. 6, SNAP-25 cleavage mediated by BoNT/E or BoNT/A separately induced a single positive signal with the corresponding mAb probes and neither of the monoclonal antibodies showed any binding in the absence of neurotoxins. The signal level for BoNT/A activity detection was also in agreement with the previously published sensitivity^20^. As shown in the last panel of Fig. 6, the use of moderate concentrations of BoNT/A and E allowed simultaneous cleavage and sequential detection, with similar signal levels, of both neurotoxins. Thus, these results indicate that our assay provides an efficient tool for consecutive and specific detection of BoNT/A and E activities on the same biosensor chip.

## Discussion

Highly sensitive, target-specific sensors are needed for identifying biological threats in health care, security, and food safety industries. We have developed an optical SPR biochip to detect and quantify BoNT/E, one of the most potent toxins affecting humans. Although optical biosensors are widely used to measure biomolecular interactions, the protocol presented in this work does not detect directly the presence of BoNT/E but a consequence of its interaction with the immobilized substrate. Indeed we have taken advantage of the enzymatic activity driven by the light chain of BoNT/E and its molecular selectivity for SNAP25 to generate a unique cleavage product on the chip. The method provides a high degree of specificity since positivity is a consequence of three distinct consecutive reactions: substrate recognition, endoproteolytic cleavage occurring at a single defined peptide bond and finally specific antibody recognition of the cleavage product. The SPR-assay is very simple, composed of two automated steps consisting in BoNT/E injection and mAb detection. The assay format with one interactant captured on a solid support mimics physiological conditions where SNAP25 is associated with the inner leaflet of the plasma membrane and cleaved by BoNT/E.

We first generated a monoclonal antibody that recognizes specifically the new C-terminal end of SNAP25 resulting from BoNT/E enzymatic activity but not cross reacting with cleavage products generated by other BoNT subtypes. This mAb provides in a minute timescale a positive gain of signal to monitor functional BoNT/E activity and constitutes a key specificity determinant of this assay. Compared to polyclonal antibodies with similar specificity^15^, our mAb should be a more robust tool, less prone to batch to batch variability. As cleavage product detection by mAb11C3 is performed in controlled buffer conditions after surface washing, the SPR assay avoids matrix interference^23^ and retains highly specific detection even when analyzing complex samples (i.e serum).

The method has been developed using biosensors equipped with either a single injection port (Biacore) or multi-ports (ProteOn). The multi-channel apparatus provides the advantage of managing several samples in parallel, and especially establishing linear plots across a range of BoNT/E concentrations. Linear calibration curves were produced in short timescales (15–30 min) for relatively high BoNT/E concentrations (1–20 LD_50_/ml) or long timescales (5h) for low concentration ranges (0.01–1 LD_50_/ml). Sensitivity is thus directly proportional to the cleavage time in a time-window between 0.25 and 5 h, but could be potentially increased providing that BoNT/E activity remains stable during an extended cleavage time.

The SPR assay offers the advantage of rapidity as BoNT/E activity at 1 LD_50_/ml can be easily detected in less than 15 minutes. Extension of the cleavage period to 5h increased detection sensitivity for BoNT/E to 0.01 LD_50_/ml, a value lower than those previously reported for BoNT/E activity-based assays^15^,^17^,^19^. As LD_50_ data can depend on assay conditions^24^ we confirmed our assay sensitivity using two different sources of BoNT/E.

We found that BoNT/E trypsinization enhances enzymatic activity as well as the toxicity in mouse. However, the activity of the trypsinized BoNT/E detected on-chip was slightly lower than what could be expected compared to data obtained from *in vivo* experiments. This observation may stem from the fact that trypsin and trypsin inhibitor present in the tryspsinized BoNT/E preparation could slightly interfere with the assay. Alternatively trypsin activation could increase not only enzymatic activity but also others factors as biodisponibility and/or cell penetration of BoNT/E, factors that are not measured in the SPR assay. This could account for the greater factor of activation with trypsinized BoNT/E determined using the mouse bioassay.

We have shown that BoNT/E could be directly detected in diluted (1:10) serum samples. A sensitivity of 0.1 LD_50_/ml was achieved in 5 hours, a concentration that was never lethal in mice. This result suggests that the SPR BoNT/E activity assay could be a faster and more sensitive method than the mouse assay. A key feature of the SPR-based assay is a rapid readout with a surface that can be probed at various times during the cleavage step. As detection of BoNT intoxication requires urgent diagnosis for timely treatment, BoNT/E activity could be evaluated using a short cleavage time (i.e. 1 hour) to detect BoNT/E concentrations ≥1 LD_50_/ml and then using more prolonged cleavage times to measure lower concentrations. Other *in vitro* methods have been developed to detect BoNT/E in serum samples. However, these tests are less convenient than the direct and automated detection method presented in this paper. Indeed, they are based on a two-step approach involving toxin concentration/purification prior to assaying toxin activity^8^,^16^,^25^,^26^.

Additionally, we have shown that this “on chip” assay can be used to detect and quantify simultaneously both BoNT/A and BoNT/E activity. This biosensor, which has similar sensitivity for both toxins^21^, could be connected to drinking water supply systems for continuous monitoring of their presence. This assay has also the potential to detect the BoNT/C activity that cleaves SNAP-25 and affects animals. Finally, this method should provide a rapid and useful tool to screen for BoNT/A and E activity inhibitors. Provided that these drugs are membrane permeable, they could be used when toxins enter the nerve cells.

## Materials and Methods

### Chemical and reagents

His_6_-SNAP-25 and mAb10F12 were prepared as described^20^. Control human sera were obtained from the “Etablissement Francais du Sang” (Marseille, France). Glutathione-S-Transferase (GST) was produced as described^27^. Amine coupling kits were from GE Healthcare and Bio-Rad. ZnCl_2_, fatty acid-free bovine serum albumin (BSA), Tween20, dithiothreitol (DTT) and Fc specific goat anti-mouse IgG were from Sigma. BoNT/A (MW 500 000 Da, 3.5 × 10^7^ LD_50_/mg), BoNT/B (MW 550 000 Da, 1.1 × 10^7^ LD_50_/mg) and BoNT/E (MW 300 000 Da, 1.5 × 10^5^ mLD_50_/mg) complex toxins at a concentration of 1 mg/ml were purchased from Metabiologics (Madison, WI). BoNT/C was provided by Prof. S. Kozaki, (Osaka Prefecture University). BoNT/E complex was prepared from culture of *C. botulinum* E strain HV in Trypticase-glucose-yeast extract broth culture at 37 °C for 4 days in anaerobic conditions^28^.

### BoNT/E trypsinization

In some cases, BoNT/E from Metabiologics was trypsin-activated by adding one part (by protein weight) of TPCK-treated trypsin (Sigma T1426) to 10 parts of toxin in 0.1 M sodium phosphate buffer pH 6.0 and incubating for 30 min at 37 °C. The reaction was stopped by adding a 10 fold excess of soybean trypsin inhibitor (Sigma T9003). The activated toxin was aliquoted and frozen. Serum samples spiked with various BoNT/E concentrations were divided in 2 parts for *in vitro* and *in vivo* assays and frozen.

### Monoclonal antibody production

Mice were immunized with a synthetic peptide corresponding to the amino acid sequence of residues 173–180 of rat SNAP-25 (GeneCust, Europe). This sequence is located immediately N-terminal to the peptide bond cleaved by BoNT/E (R_180_-I_181_). A cysteine residue was added to the N-terminus of the peptide to allow coupling to Keyhole Limpet Hemocyanin (KLH) for immunization. Hybridomas were selected by ELISA for their ability to recognize the immunization peptide and by SPR for their ability to capture SNAP-25 in plasma membrane vesicles treated with BoNT/E^29^. The selected monoclonal antibodies (mAb11C3) were affinity purified using protein A-Sepharose and stored at −20 °C.

### Preparation of SNAP-25 chips

Purified His-SNAP25 was amine coupled at pH 4 to ProteOn GLC (Bio-Rad) sensor chips or at pH 4.5 to Biacore CM5 (GE Healthcare) according to manufacturer’s instructions and phosphate-buffered saline (PBS) as running buffer. The final density was approximately 5 000 RU on GLC chips and 10 000 RU on CM5 chips. All chips were conditioned by injection of cleavage buffer (50 mM HEPES (4-(2-hydroxyethyl)-1-piperazine ethane sulfonic acid)-NaOH pH 7.4, 0.1% BSA, 5 mM DTT, 1% Tween 20, 100 μM ZnCl_2_). To determine the specificity of mAb11C3, His-SNAP25 (0.5 mg/ml) was pre-incubated for 2 h at 37 °C in cleavage buffer without BSA in presence or absence of 10 nM BoNT/E. Intact His-SNAP25, His-SNAP25 cleaved by BoNT/E, and GST were coupled (500 RU) on GLC sensor chip as mentioned before.

### SPR analysis of BoNT/E endoprotease activity

SPR measurements were performed at 34 °C using ProteOn XPR36 (Bio-Rad) or Biacore T200 (GE Healthcare) apparatus and TBS (10 mM Tris-HCl pH 7.4, 150 mM NaCl) as running buffer. Flow rates were 5 and 25 μl/min, with the Biacore T200 and ProteonXPR36 apparatus respectively. The ProteOn XPR36 has six parallel flow channels that can be used to uniformly immobilize strips of six ligands on the sensor surface. The fluidic system can automatically rotate 90° so that up to six different analytes can be injected, allowing simultaneous monitoring of up to 36 individual molecular interactions in a single run on a single chip (Bravman *et al*., 2006). Data were analyzed using Biaevaluation 4.2 software (GEHealthcare) or ProteOn Manager v. 3.1.0.6 software (Bio-Rad).

BoNT/A, B, C and E (trypsinized or not) were thawed and serially diluted in cleavage buffer. Serially diluted BoNT/E samples and mAb11C3 (10 μg/ml) were placed in the sample rack at 4 °C before injection over the His-SNAP25 coated chip. BoNT/E samples were injected using different contact times (cleavage step) whereas mAb11C3 was injected for 2 min over all flow cells to measure cleaved SNAP-25 (detection step). In experiments with signal amplification, anti-mouse Fc antibodies were diluted at 10 μg/ml and injected (for 4 minutes) immediately after mAb11C3 (co-inject protocol). Antibody binding was measured in triplicate, 10 sec after the end of injection(s) and the chip was regenerated with a 8 sec pulse (100 μl/min) of 10 mM glycine-HCl pH 2.0. In all experiments, non-specific signals were measured by injecting cleavage buffer without BoNT onto a flow cell coated with His-SNAP25. Data obtained from control flow cells were automatically subtracted from experimental measurements to yield the specific signal. Limits of detection (LODs) were calculated by determining the minimal BoNT concentration producing a signal 3 standard deviations (SDs) higher than the control values in the absence of BoNT (*n* = 6). For BoNT/A and BoNT/E co-detection experiments, consecutive co-inject protocols using mAb11C3 (10 μg/ml)/anti-mouse Fc antibody and mAb10F12/anti-mouse Fc antibody injections were separated by a pH 2 regeneration step.

### *In vivo* assay

Mice were injected intraperitoneally with 1 ml of serially diluted BoNT/E serum as described 30. Symptoms of botulism were monitored for 4 days. Experiments were performed in accordance with French and European community guidelines for handling laboratory animals. The protocols of experiments were approved by Pasteur Institute CETEA (Comité d’Ethique en Expérimentation Animale) with the agreement of laboratory animal use (n° 2013-0118).

## Additional Information

**How to cite this article**: Lévêque, C. *et al*. An optical biosensor assay for rapid dual detection of Botulinum neurotoxins A and E. *Sci. Rep*. **5**, 17953; doi: 10.1038/srep17953 (2015).

## Supplementary Material

Supplementary Information

## Figures and Tables

**Table 1:** Measurements of BoNT/E activity by the SPR-based assay.

	BoNT/E			Trypsinized BoNT/E	
Cleavage time	5 min	15 min	5 h	15 min	5 h
LD_50_/ml	1	1	0.01	1	0.03
Detection (RU)	8.6 ± 1.3* (n = 3)	16.3 ± 2.7* (n = 5)	10.4 ± 1.4 (n = 4)	20.1 ± 5.0 (n = 5)	4.1 ± 1.3 (n = 3)

All experiments were performed on a ProteOnXPR36 biosensor except the experiment with 5 min cleavage time that was carried out on a Biacore T200. *Represents mAb11C3 binding without amplification. The LOD values are 1.5 RU for the Biacore T200 versus 1.9 RU (non-amplified) and 0.8 RU (amplified) for the ProteOnXPR36.

**Table 2:** Comparison of the SPR assay and the mouse bioassay for serum sample analysis.

Estimated LD50/mouse	SPR assay mAb bound (RU)	Mouse bioassay (n° of deaths/total n° of mice)
5	366.0 ± 9.4 (n = 1)	9/9
1	156.6 ± 28.9 (n = 3)	8/13
0.5	89.6 ± 12.4 (n = 3)	0/5
0.25	54.7 ± 6.8 (n = 2)	ND
0.125	20.7 ± 8.0 (n = 2)	ND
0	1.6 ± 2.5 (n = 3)	ND

BoNT/E was diluted in serum and aliquoted before in vitro and in vivo measurements. SPR experiments were repeated independently 2 or 3 times for BoNT/E concentrations =1 LD50/ml after 10 fold dilution in different sera (LOD value = 9.2 RU). Mice were observed for 4 days. ND, not done.
