# Supplementary material for: An optical biosensor assay for rapid dual detection of Botulinum neurotoxins A and E
Source: Sci Rep. 2015 Dec 9;5:17953. doi: 10.1038/srep17953 (PMC4673697; doi:10.1038/srep17953)
Supplement: Supplementary Information [file srep17953-s1.pdf]

An optical biosensor assay for rapid dual detection of Botulinum neurotoxins A and E

Christian Lévêque[1,3,+], Géraldine Ferracci[2,3,+], Yves Maulet [1,3], Christelle Mazuet[4],

Michel R. Popoff[4], Marie-Pierre Blanchard [2,3], Michael Seagar[1,3], and Oussama El Far [1,3,*]

1. INSERM, UMR_S 1072, 13015 Marseille, France
2. CNRS, UMR 7286, Plate-Forme de Recherche en Neurosciences PFRN, 13015 Marseille, France
3. Aix-Marseille Université, 13015 Marseille, France
4. CNR Bactéries Anaérobies et botulisme, Unité des Bactéries anaérobies et toxines. Institut Pasteur, 28 rue du Dr Roux, 75724 Paris Cedex 15, France
* oussama.el-far @inserm.fr
[+]these authors contributed equally to this work

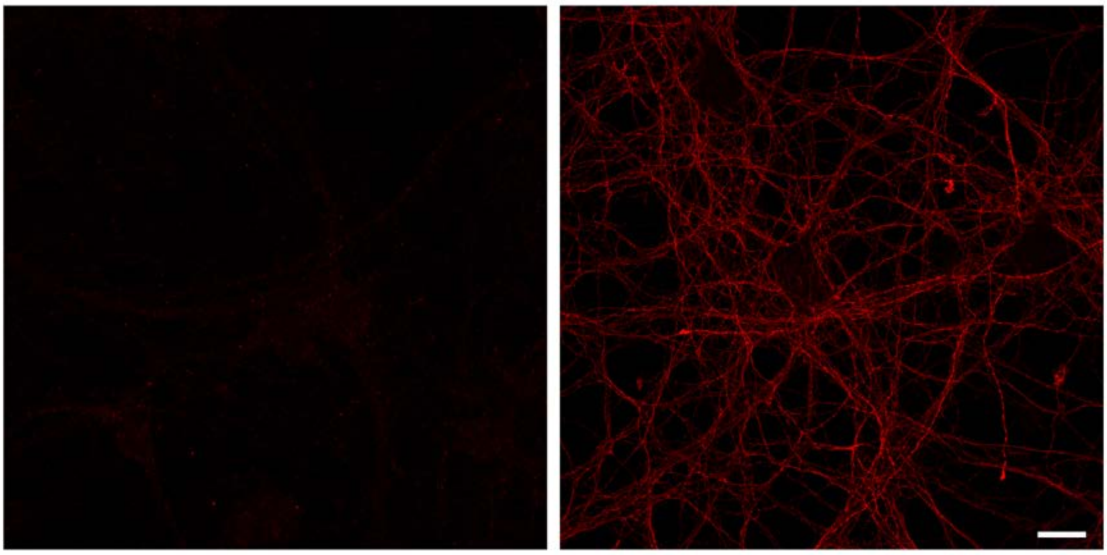

Figure S1: Detection of SNAP25 cleaved by BoNT/E in dissociated cultured hippocampal neurons.

Cultured rat hippocampal neurons were prepared as described (Ferracci et al., 2011). Cells at DIV 15 were either untreated (control) or exposed to BoNT/E (1nM) for 4 hours. Cells were then fixed for immunochemistry. SNAP-25 cleavage was detected using mAb11C3. Scale bar = 20 µm

Ferracci, G. *et al.* A label-free biosensor assay for botulinum neurotoxin B in food and human serum. *Anal Biochem* 410, 281-288, doi:10.1016/j.ab.2010.11.045 (2011)

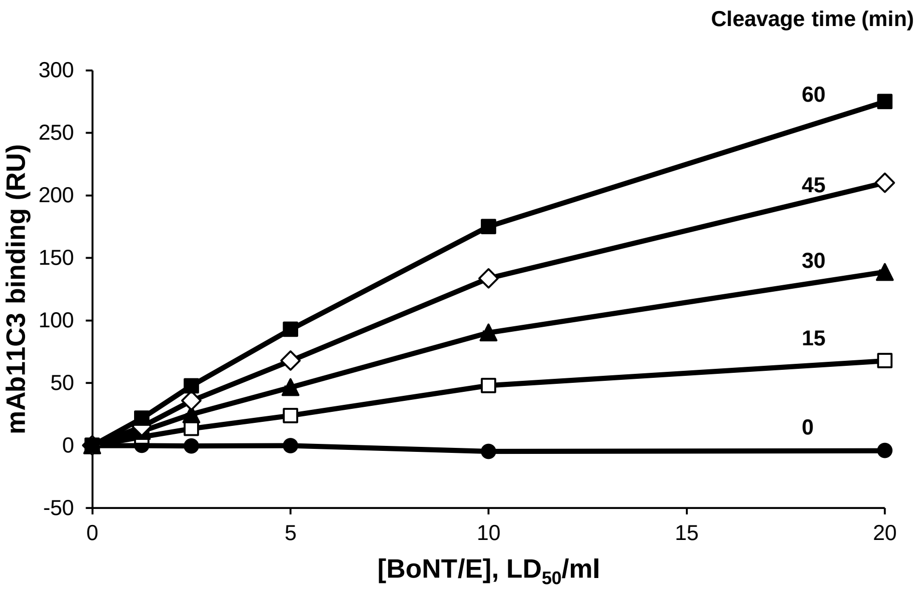

Figure S2: Loss of linearity in the measurement of SNAP-25 cleavage using high BoNT/E concentrations.

Samples with various BoNT/E concentrations (0, 1.25, 2.5, 5, 10, 20 LD_{50}/ml) were injected for 4 sequential 15 min periods (ie. to yield 15, 30, 45 and 60 min cumulated cleavage times) over immobilized SNAP-25. At the end of each 15 min injection, detection of BoNT/E enzymatic activity was achieved by injecting mAb11C3. Data represent dose-response curves for each cleavage time. Notice the loss in linearity above 10 LD_{50}/ml.
